# Supplementary material for: Self-Assembled CuCo2S4 Nanoparticles for Efficient Chemo-Photothermal Therapy of Arterial Inflammation
Source: Molecules. 2022 Nov 22;27(23):8134. doi: 10.3390/molecules27238134 (PMC9737671; doi:10.3390/molecules27238134)
Supplement: Supplementary file 1 [file molecules-27-08134-s001.zip › molecules-2023693-supplementary.pdf]

Chloroquine-Loaded Self-Assembled $CuCo_2S_4$ Nanocrystals for Efficient Chemo-Photothermal Therapy of Arterial Inflammation

Ran Lu [1,†], Wei Wang [2,†], Bo Dong [2], Chao Xu [1], Bo Li [3], Yong Sun [1,*], Junchao Liu [3] and Biao Hong [2,*]

[1] Department of Vascular Surgery, The First Affiliated Hospital of Bengbu Medical College, Bengbu 233004, China
[2] Department of Vascular Surgery, Tongren Hospital, Shanghai Jiao Tong University School of Medicine, Shanghai 200336, China
[3] Department of Vascular Surgery, Shanghai Ninth People's Hospital, Shanghai Jiao Tong University School of Medicine, Shanghai 200011, China
* Correspondence: sunyong197708@163.com (Y.S.); HB2811@shtrhospital.com (B.H.)
† These authors contributed equally to this work.

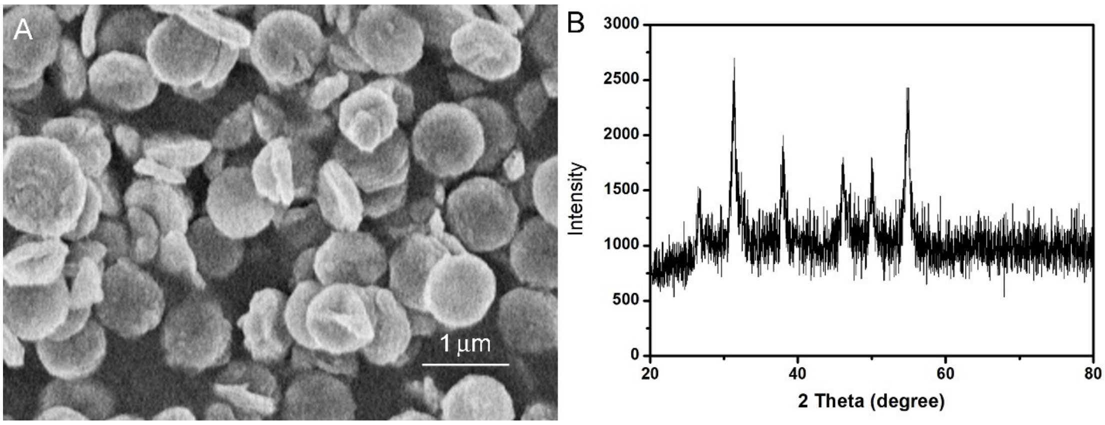

Figure S1. Characterization of self-assembled CuCo2S4 NCs. (**A**) SEM image of the self-assembled CuCo2S4 NCs. Scale bar = 1 μm. (**B**) Powder XRD patterns of the self-assembled CuCo2S4 NCs.

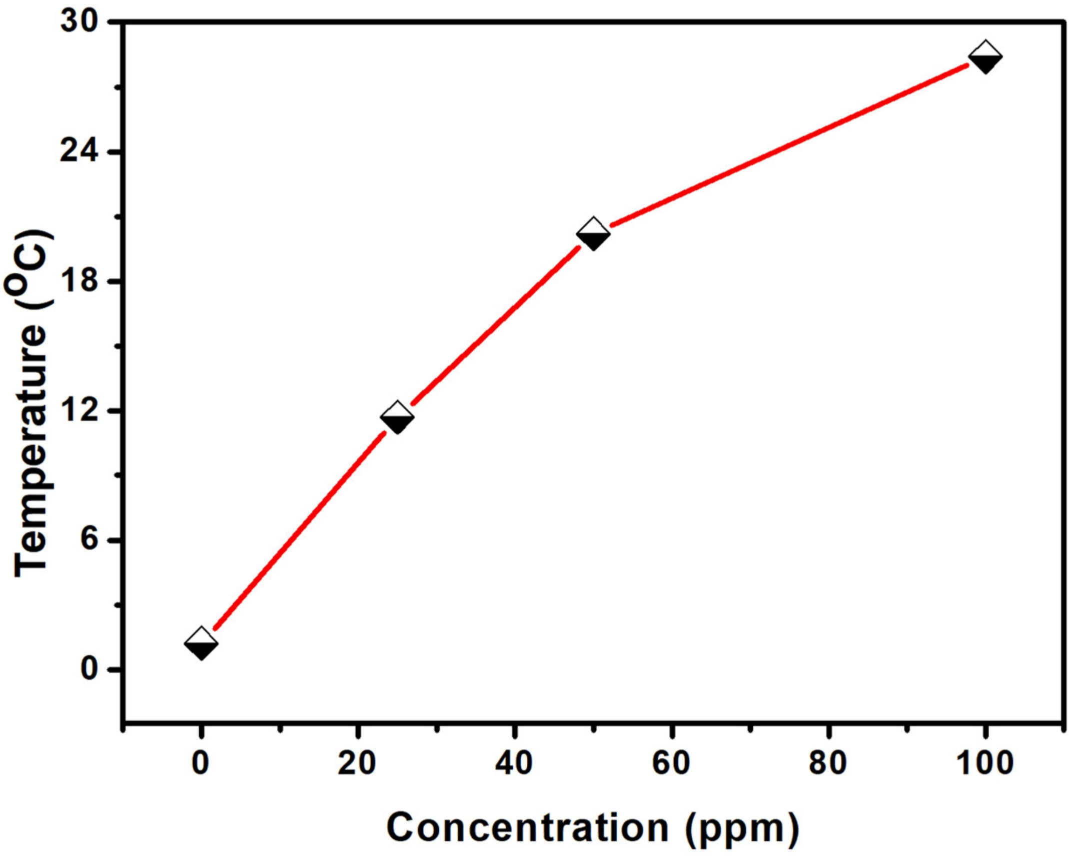

Figure S2. The relationship between temperature and concentration of self-assembled CuCo2S4 NCs.

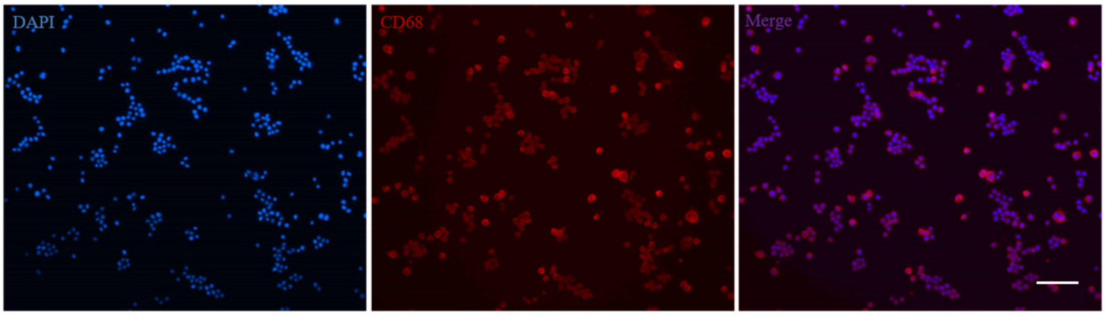

Figure S3. Immunofluorescence of Raw264.7 macrophages stained with DAPI (blue), CD68 (red), and both. Scale bar = 100 μm.

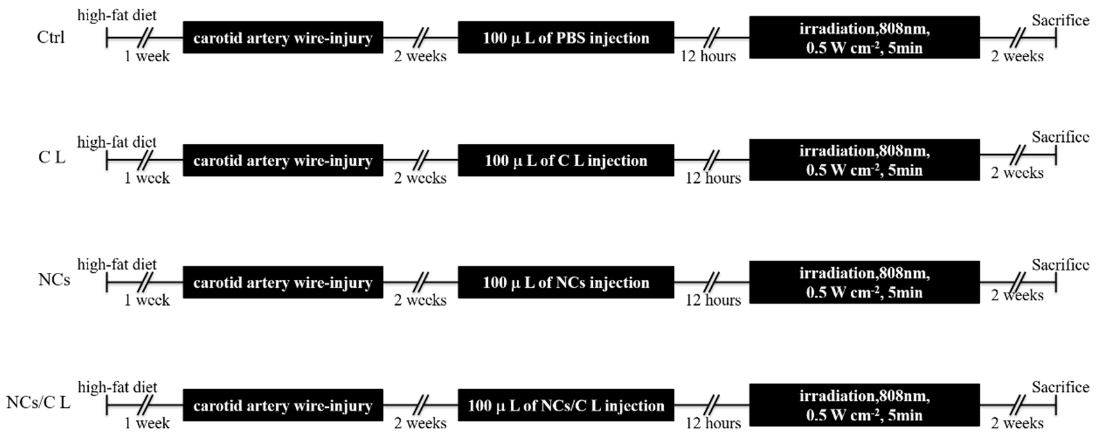

Figure S4. Flow diagram of in vivo experiments.
